# Epidemiological characterization of avian influenza virus circulation in pigeons and quail across different production systems in Chattogram, Bangladesh

**DOI:** 10.1371/journal.pone.0361561

**Published:** 2026-10-26

**Authors:** Mohammad Iqbal Habib, Md Abu Sayeed, Monjurul Islam, Mohammad Ferdous Rahman Sarker, Sydus Shalekin Shakil, Mohammad Mahmudul Hassan, Jade K. Forwood, Abdul Ahad, Ariful Islam

**Affiliations:** 1 Faculty of Veterinary Medicine, Chattogram Veterinary and Animal Sciences University, Chattogram, Bangladesh; 2 Zoonotic Disease Research Program, Institute of Epidemiology, Disease Control and Research (IEDCR), Mohakhali, Dhaka, Bangladesh; 3 National Centre for Epidemiology and Population Health (NCEPH), Australian National University, Canberra, Australia; 4 Gulbali Research Institute, Charles Sturt University, Wagga Wagga, Australia; 5 Queensland Alliance for One Health Sciences, School of Veterinary Science, The University of Queensland, Gatton, Australia; Sudan University of Science and Technology, College of Veterinary Medicine, SUDAN

## Abstract

Pigeons and quail contribute to poultry production, nutrition, and livelihoods in Bangladesh; however, their role in avian influenza virus (AIV) circulation remains poorly understood. These poultry species are widely reared and traded alongside chickens and ducks in household farms, commercial systems, and live bird markets, where inadequate biosecurity may create an overlooked ecological niche for AIV circulation, reassortment, and potential zoonotic spillover. Between November 2021 and April 2022, we conducted a cross-sectional study in Chattogram, Bangladesh, to estimate the prevalence and subtype-specific distribution of AIV and to identify factors associated with AIV positivity in pigeons and quail. A total of 300 birds (108 pigeons and 192 quail) were sampled from households, commercial farms, and live bird markets. Oropharyngeal and cloacal swabs were collected from each bird and tested for the AIV matrix gene using real-time reverse transcription polymerase chain reaction, followed by subtype-specific detection of H5, H7, and H9. Data collected from bird owners and traders included bird demographic characteristics, bird source, and health status using a structured questionnaire. The overall prevalence of AIV was 14.3% (95% confidence interval [CI]: 10.6–18.8). Quail had a higher prevalence (18.2%, 95% CI: 13.0–24.4) than pigeons (7.4%, 95% CI: 3.3–14.1). Among detected subtypes, H9 was most prevalent (11.3%, 95% CI: 7.9–15.4), followed by hemagglutinin (HA)-untyped (2.7%, 95% CI: 1.2–5.1) and H5 (0.3%, 95% CI: 0.001–1.8), while H7 was not detected. Quail had higher odds of AIV positivity than pigeons (adjusted odds ratio [aOR] = 4.1, 95% CI: 1.8–10.6); clinically sick birds (aOR = 2.9, 95% CI: 1.4–6.2) and birds sampled from household farms (aOR = 2.8, 95% CI: 1.2–6.5) also had higher odds of AIV positivity. These findings indicate that pigeons and quails, which are understudied poultry species, may contribute to the maintenance and transmission of AIV within mixed poultry production and marketing systems in Bangladesh. Integrating these species into routine AIV surveillance is therefore necessary to strengthen early detection, inform control strategies, and reduce the risk of zoonotic spillover.

## Introduction

Avian influenza (AI), caused by the Avian Influenza Virus (AIV), remains a serious threat to poultry production, global food security, economy, and public health. AIV is a member of the *Orthomyxoviridae* family and has negative-sense, single-stranded, segmented RNA that can infect not only birds but also a wide range of mammals, including humans, pigs, and horses, making it a significant public health concern [1–4]. Wild waterfowl function as natural reservoirs for AIV that facilitate the emergence of novel strains and sustain both pathogenicity and transmission, which underscores the necessity to understand AIV’s reservoirs and transmission dynamics [5,6].

Migratory wild waterfowl may connect distant ecosystems to new regions and introduce new viral strains into vulnerable domestic and wild populations [7]. The intermingling of wild birds with domestic and farmed poultry in agroecological settings further increases the possible risk of AIV spillover [8]. Monitoring migration routes and inter-species contact networks is therefore essential to anticipate and prevent AI outbreaks [9]. Importantly, recent outbreaks have been dominated by the emergence and the global spread of pandemic potential high pathogenicity avian influenza (HPAI) clade 2.3.4.4b, which is responsible for unprecedented mortality in poultry and wild birds [10].

The transmission of AIV is shaped not only by ecological factors but also by human activities like the establishment of poultry farms near wetlands or migratory flyways that may act as potential hotspots for viral amplification and spillover [11]. Additionally, poor biosecurity in live bird markets (LBMs) are responsible for creating ideal conditions for AIV to persist and spillover to humans and wildlife [12]. Similarly, inadequate farm biosecurity, including unrestricted visitor access and wild bird intrusion, may further amplify transmission risks across animal reservoirs and into human populations [13,14]. Therefore, strengthening biosecurity practices and addressing these anthropogenic drivers are crucial to reducing the risk of AIV spillovers to both poultry and public health.

AIV are classified based on their pathogenic potential, where the HPAI’s different subtypes, like H5N1 and H7N9, are responsible for severe disease and high mortality, while low pathogenicity avian influenza (LPAI) typically produces milder disease symptoms [15–17]. Though AIV has sixteen HA (H1–H16) and nine NA (N1–N9) antigenic variations [18] they can evolve through their antigenic drift and shift mechanism to adapt to a new host species and fuel outbreaks [19]. So, the ability of LPAI to evolve into HPAI remains a major concern for poultry health and production, making it essential to understand the genetic adaptability of AIV to predict and control future outbreaks. This adaptability is also responsible for the clade diversification that has led to the emergence of multiple H5Nx subtypes globally through reassorting viruses with heightened zoonotic potential [10].

The history of the devastating outbreak of HPAI H5N1 in Bangladesh illustrates its escalating impact, which started with the emergence of AI outbreaks in China in 1996 and then spread across South Asia [20,21]. Repeated waves of AI outbreaks have not only posed zoonotic risks but also caused heavy economic losses, particularly in broiler and layer production [22]. Household poultry farms have also been identified as important risk factors for spreading HPAI H5N1 to commercial farms [23]. Surveillance has documented the circulation of multiple clades, including 2.2.2, 2.3.2, 2.3.2.1a, and more recently 2.3.4.4b, of H5N1 in Bangladesh, which highlights the dynamic nature of HPAI evolution and cross-border transmission via migratory birds and trade [10]. Additionally, seasonal patterns tied to migratory birds together with inadequate biosecurity further sustain the endemic status of AI in Bangladesh [24]. The co-circulation of HPAI H5N1 and LPAI H9N2 in poultry raises concern about possible reassortment events that could generate novel viruses with increased zoonotic and pandemic potential [25].

While wild and domestic birds have been thoroughly studied in this context, quail and pigeons remain overlooked. Bangladesh exemplifies the urgency of addressing this gap due to its dense poultry populations, bustling LBMs, and widespread quail and pigeon population. Although past research has documented shifts in HPAI H5N1 clades, it has not adequately addressed the risks associated with pigeons and quails [26].

In Bangladesh, both pigeon and quail production have shifted from traditional backyard or pet bird keeping towards increasingly commercial production systems in response to the growing demand for alternative poultry meat and eggs. Pigeons, with a national population exceeding 10 million, are valued for their prolific reproduction and the delicacy of squab meat, offering income-generation opportunities for rural communities [27]. Similarly, quails are now commercially farmed for both meat and egg production because their meat and eggs contain high protein and they possess fast growth and strong reproductive efficiency [28]. So, their presence in LBMs has increased as demand has grown, but most vendors still have limited knowledge about biosecurity. Considering that pigeons and quails increasingly share production, trading, and marketing environments with other poultry species, they may be exposed to similar opportunities for AIV introduction, maintenance, and transmission. Despite their growing importance in Bangladesh’s poultry sector, their epidemiological role remains poorly understood compared with chickens and ducks [29]. Therefore, understanding AIV circulation in these two understudied poultry species is essential for strengthening surveillance and informing effective control strategies.

Quail and pigeons can serve as reservoirs for H9N2, posing considerable economic challenges for poultry farming by complicating efforts to control virus circulation within commercial poultry [30]. Although classified as low pathogenic, H9N2 can cause reduced egg production and impaired growth rates in infected flocks, which leads to substantial economic losses among poultry farmers [31,32]. Although vaccines have been developed for poultry, their effectiveness is often limited due to species-specific variations in immune response and viral replication dynamics [32]. Consequently, the existing vaccination strategies do not adequately address the challenges posed by H9N2 infection in quails, highlighting the need to reassess vaccine efficacy and broaden vaccination strategies [29].

Given the expanding commercial production and shared marketing of pigeons and quails, the present study aimed to estimate the prevalence and subtype distribution of AIV in these species across households, commercial farms, and LBMs in Chattogram, Bangladesh. In addition, this study sought to identify factors associated with AIV prevalence in these poultry species, generating evidence to inform control strategies and support efforts to protect both animal and human health.

## Materials and methods

### Study location, design, and duration

This study was conducted in the Chattogram district, located in the southeastern part of Bangladesh (22.130–22.270° N, 91.400–91.530° E). Chattogram is the second largest city in the country and a principal maritime hub, covering approximately 157 km² and characterized by a diverse landscape that includes hills, rivers, and low- and high-lying areas. The district was selected because it has one of the country’s largest poultry production and marketing networks, with extensive household and commercial pigeon and quail production and numerous LBMs. We conducted this cross-sectional study to investigate AIV prevalence in pigeons and quails from November 2021 to April 2022. The study involved structured questionnaire surveys and the collection of biological samples. The Chattogram district comprises 15 subdistricts (known as upazilas). To ensure an adequate sampling frame across all production systems of interest, we included nine of the 15 upazilas (Chattogram Metropolitan City, Patiya, Chandanaish, Satkania, Lohagara, Anwara, Banshkhali, Hathazari, and Boalkhali). These upazilas were selected because they had active pigeon and quail production systems and live bird marketing activities, including household farms, commercial farms, and LBMs. The spatial distribution of the study area and sampling sites is illustrated in the digitized map of Bangladesh using QGIS v3.42.3 (https://qgis.org/) (Fig 1).

**Fig 1:**
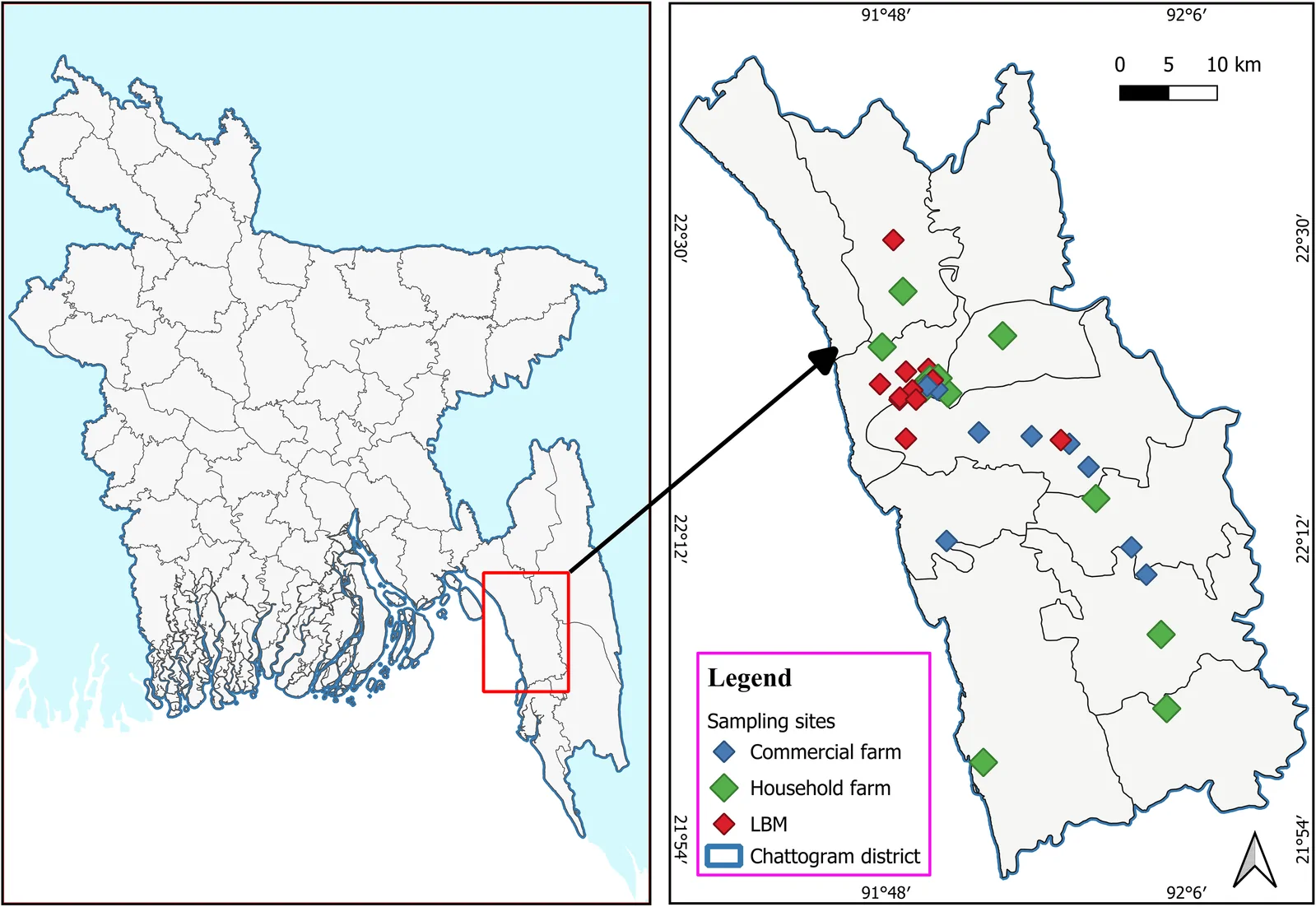
Spatial location of sampling sites; Blue diamond indicates location of sampled commercial farm, green diamond indicates location of sampled household farm, and red diamond indicate location of sampled LBM. The map was created using a shapefile from the freely accessible GADM database (GADM; www.gadm.org) [33].

### Study population, sampling frame, and sample size

The study population consisted of households, commercial farms, and LBMs involved in the raising and selling of pigeons and quail. Initially, we developed a comprehensive list of households and commercial farms raising pigeons and quail, along with LBMs having stalls with at least 10 birds, located in the selected subdistrict of the Chattogram region. From the sampling frame, we randomly recruited a list of 11 households, 10 commercial farms, and 11 LBMs for inclusion in the study. The number of study sites within each production system reflected the availability of eligible pigeon and quail populations in the sampling frame and field feasibility while ensuring representation of all three production systems. A total of 300 birds were randomly selected, comprising 192 quails and 108 pigeons. The sample size was determined based on the expected prevalence of AIV, considering 17.3% in pigeons and 38.7% in quails, aiming for a 95% confidence level and a precision of ±7%. The required sample sizes to achieve this precision were approximately 113 for pigeons and 186 for quails. The collected samples (108 pigeons and 192 quails) were thus sufficient to achieve close to the desired precision for pigeons and exceeded the requirement for quails. The sample size calculations were performed using the OpenEpi online tool (http://www.openepi.com/Menu/OE_Menu.htm), as described by Sayeed et al. [12]. A precision level of ±7% was selected to ensure reliable estimates while maintaining feasibility under field conditions [34].

### Ethical approval

This study involved both animal sampling and the collection of questionnaire data from bird owners and market traders. All animal procedures were conducted in accordance with institutional guidelines for the ethical use and welfare of animals in research. Biological swab samples were collected from pigeons and quails by trained field veterinarians using appropriate physical restraint and handling techniques to minimize stress and ensure the welfare of the birds throughout the sampling process.

The study objectives and procedures were clearly explained to bird owners and market traders, and written informed consent was obtained from all participants prior to sampling farmed birds and birds from LBMs.

At Chattogram Veterinary and Animal Sciences University (CVASU), ethical review is conducted under a single institutional Ethics Committee (EC) comprising independent expertise in animal ethics and welfare, human-participant research ethics, and environmental sciences. Research protocols are reviewed by the relevant ethical component(s) according to the nature of the proposed work. Accordingly, the animal-sampling procedures in this study were reviewed for animal welfare and ethical compliance, while the questionnaire-based component involving bird owners and market traders was reviewed for human-participant considerations. Final institutional approval for the complete study protocol was issued by the Ethics Committee of CVASU under Memo No. CVASU/Dir(R&E)EC/2019/126(1), dated 29 December 2019. All biological samples and study data were collected prospectively following ethical approval.

For the human-participant component only, the study was conducted in accordance with the ethical principles of the Declaration of Helsinki. No human biological specimens were collected.

### Biological sample and data collection, transportation, and storage

Cloacal and oropharyngeal swabs were collected from each bird. Cloacal swabs were obtained by gently inserting a sterile polyester-tipped applicator (Puritan®, model: REF 25–801 D50, USA) into the vent (cloacal region) until fecal material was visible. Oropharyngeal swabs were taken by swabbing the oropharyngeal wall and airway. From each bird, the subsequent cloacal and oropharyngeal swabs were combined in a single 1.8 mL sterile cryovial containing 1.0 mL of viral transport medium (VTM) [35]. A total of 300 birds were randomly sampled from three types of bird sources. Swab samples were collected from 98 birds (68 quail and 30 pigeons) from 11 LBMs, 134 birds (91 quail and 43 pigeons) from 10 commercial farms, and 68 birds (33 quail and 35 pigeons) from 11 household farms. Each cryovial was labelled with a unique identifying number. Immediately after collection, samples were placed in insulated ice boxes to maintain the cold chain and transported to the laboratory within 4–5 hours. In the laboratory, the samples were stored at –80°C until laboratory analysis.

In parallel with biological sampling, information on bird health status and management practices was collected from bird owners and traders using structured questionnaires, informal interviews, and observation checklists. The age of each bird was obtained from the owner or farm records, where available, at the time of sample collection and was subsequently categorized as <6 months, 6–12 months, or >1 year for analysis. All birds were clinically assessed by a licensed veterinarian prior to sample collection and classified as clinically healthy or sick based on the presence or absence of observable clinical signs, including respiratory distress, nasal discharge, depression, or reduced activity. All samples were collected wearing appropriate personal protective equipment (PPE) to ensure the safety of both the birds and the handlers.

The study involved the collection of biological specimens from pigeons and quail and did not include the collection of human biological samples. Human interaction was limited to obtaining bird-level and farm-level information and conducting field observations at households, LBMs, and agricultural settings. No personal, sensitive, or identifiable human data were collected. Written consent for participation and bird sampling was obtained from all owners and traders in accordance with the approved ethical protocol.

### Laboratory testing

In the laboratory, samples from six individual birds were combined to create one pooled sample, resulting in 50 pooled samples that were initially prepared for viral RNA extraction and AIV M-gene detection. Viral RNA was extracted from each pool using the MagMAX™-96 AI/ND Viral RNA Isolation Kit (Applied Biosystems San Francisco, CA) on a KingFisher™ Flex 96-well system following the manufacturer’s instructions. Initially, pooled swab samples were screened for the AIV M gene by real-time reverse transcription PCR (rRT-PCR) using standard primers and probes [36,37].

For any pool that tested positive for the AIV M-gene, RNA was subsequently extracted from the corresponding individual bird samples, and each individual sample was re-tested for the M gene by rRT-PCR. Birds were classified as AIV-positive if their individual swabs tested positive for the M-gene, and those from negative pools containing individual samples were classified as AIV-negative. Individual AIV-positive samples were then tested to identify specific subtypes using H5, H7, and H9 hemagglutinin gene-specific primers and probes following established protocol [37,38]. Samples that were positive for AIV RNA (M-gene) but negative for H5, H7, and H9 subtypes were classified as HA-untyped [27].

### Statistical analysis

Field and laboratory data were recorded in Microsoft Excel, and statistical analyses were performed using R version 4.3.2 (R Foundation for Statistical Computing, Vienna, Austria). We performed descriptive analysis based on rRT-PCR results to estimate the prevalence of AIV and H5, H7, and H9 subtypes across household farms, commercial farms, and LBMs, as well as their spatial distribution across the study areas. Analysis of factors associated with AIV positivity included both univariate and multivariable logistic regression approaches. In the univariate analysis, associations between AIV positivity and factors such as species, age, sex, health condition, and bird source were assessed using Fisher’s Exact test. For statistical analyses, species were categorized as pigeons or quails; age was grouped as <6 months, 6–12 months, or >1 year; sex as male or female; health condition as healthy or sick; and bird source as household farms, commercial farms, or LBMs. Variables with p < 0.10 in the univariate analysis were considered candidate variables for inclusion in the multivariable logistic regression model; this threshold was used solely for candidate-variable screening, consistent with recommended variable selection approaches for multivariable regression modelling [39]. Multicollinearity among explanatory variables was assessed using Cramer’s V. When two variables had strong collinearity (Cramer’s V > 0.4), one of the variables was excluded from the final model. The remaining variables were retained in the model to estimate adjusted associations with AIV positivity. Possible interaction terms among the explanatory variables were assessed, and no statistically significant interactions were identified. Regression model outputs were reported as adjusted odds ratios (aORs) with 95% confidence intervals (CIs) and corresponding p-values, with statistical significance set at p < 0.05.

## Results

### Prevalence and subtype distribution of AIV in pigeon and quail

Overall, the prevalence of AIV was 14.3%. Among the detected subtypes, H9 was the most prevalent (11.3%), followed by HA-untyped (2.7%). In quails, the overall AIV prevalence was higher at 18.2%, compared to 7.4% in pigeons. The prevalence of H9 specifically was 14.6% in quails and 5.6% in pigeons (Table 1).

**Table 1:** Overall AIV and subtype-specific (H5, H7, H9) prevalence in poultry species.

Variable	Category (N)	AI n (%, 95% CI)	H5 n (%, 95% CI)	H7 n (%, 95% CI)	H9 n (%, 95% CI)	HA-untyped n (%, 95% CI)
Overall	(N = 300)	43 (14.3, 10.6–18.8)	1 (0.3, 0.0001–1.8)	0	34 (11.3, 7.9–15.4)	8 (2.7, 1.2–5.1)
Species	Quails (n = 192)	35 (18.2, 13.0–24.4)	0	0	28 (14.6, 9.9–20.4)	7 (3.7, 1.5–7.4)
	Pigeons (n = 108)	8 (7.4, 3.3–14.1)	1 (0.9, 0.0002–5.1)	0	6 (5.6, 2.1–11.7)	1 (0.9, 0.02–5.1)

### Prevalence of AIV and its subtypes in quail by host and management factors

Among quails, the highest prevalence of both AIV and H9 was observed in the Japanese breed, female birds, and clinically sick birds (Fig 2). Overall, AIV prevalence was highest in Japanese quail breed (17.1%, 95% CI: 12.1–23.2), females (13.5%, 95% CI: 9–19.2), sick birds (12.5%, 95% CI: 8.1–18), and birds aged 1–3 months (9.9%, 95% CI: 6–15). Similarly, H9 prevalence was highest in the Japanese breed (13.5%, 95% CI: 9–19.2), females (10.9%, 95% CI: 6.9–16.2), and sick birds (9.3%, 95% CI: 5.6–14.4). In contrast, the Bob white breed, healthy birds, and males consistently showed lower prevalence of both AIV and H9, while HA-untyped virus detections remained low across all categories (Fig 2).

**Fig 2:**
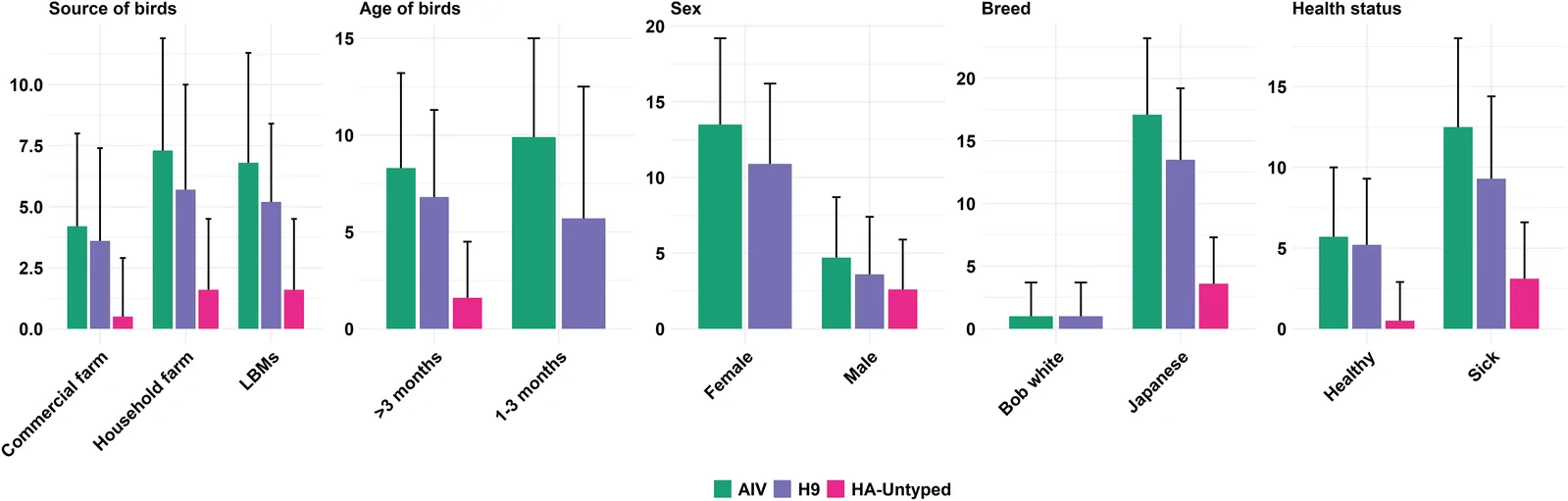
Prevalence of AIV and subtypes (H5, H9, HA-untyped) with 95% confidence intervals among quails by host and management factors.

### Prevalence of AIV and its subtypes in pigeons by factors

Among pigeons, the highest prevalence of AIV was observed in birds sourced from LBMs (4.6%, 95% CI: 1.5–10.5), birds older than one year (5.5%, 95% CI: 2.1–11.7), males (4.6%, 95% CI: 1.5–10.5), Deshi breed (6.4%, 95% CI: 2.6–12.8), and sick birds (5.5%, 95% CI: 2.1–11.7). Similarly, H9 prevalence was highest in Deshi pigeons (5.5%, 95% CI: 2.1–11.7), sick birds (5.4%, 95% CI: 1.5–10.5), and those from LBMs (4.6%, 95% CI: 1.5–10.5). In contrast, pigeons from commercial farms, healthy birds, and Kormona breeds showed a notably lower prevalence of both AIV and H9 (Fig 3).

**Fig 3:**
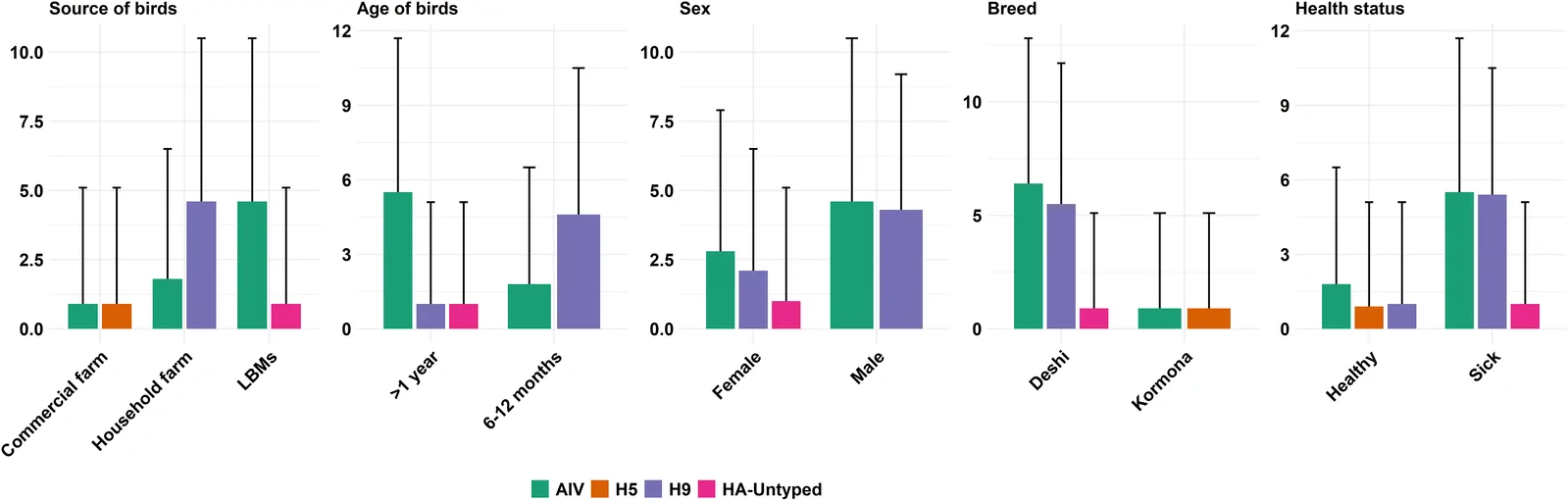
Prevalence of AIV and subtypes (H5, H9, HA-untyped) with 95% confidence intervals among pigeons by different factors.

### Factors associated with AIV positivity in pigeons and quail

#### Univariable analysis.

In univariable analysis, AIV positivity differed across several factors, including bird species, age, health condition, and bird source, meeting the prespecified screening criterion of p < 0.10 and was considered a candidate variable for multivariable analysis. Quail had a higher AIV positivity than pigeons. Birds younger than 6 months showed the highest AIV positivity. Sick birds had a higher positivity compared to healthy birds (Table 2).

**Table 2:** Univariable associations of AIV positivity with selected factors in pigeons and quails.

Variable	Category	N	Positive (%)	95% CI	p-value
Species	Pigeons	108	8 (7.4)	3.3-14.1	0.01*
	Quails	192	35 (18.2)	13-24.4	
Age	< 6 months	120	23 (19.1)	12.6-27.4	0.07*
	6-12 months	102	14 (13.7)	7.7-22	
	> 1 year	78	6 (7.6)	2.9-16	
Sex	Female	203	29 (14.2)	9.8-19.9	0.97
	Male	97	14 (14.4)	8.1-23	
Health condition	Healthy	159	13 (8.1)	4.4-13.6	0.01*
	Sick	141	30 (21.2)	14.8-29	
Source of sample	LBMs	98	15 (15.3)	8.8-24	<0.01*
	Commercial Farms	134	9 (6.7)	3.1-12.4	
	Household Farms	68	19 (27.9)	17.7-40.1	

* p-value < 0.10; statistically significant

#### Multivariable logistic regression analysis.

Strong collinearity was observed between species and age (Cramer’s V = 0.8); therefore, age was excluded from the multivariable model, while species was retained as a primary explanatory variable of interest. The final multivariable model included species, health condition, and bird source. No statistically significant interactions were identified among the variables assessed.

Quails had significantly higher odds of AIV positivity than pigeons (aOR = 4.1, 95% CI: 1.8–10.6, p < 0.01). Clinically sick birds were nearly three times more likely to test positive compared to apparently healthy birds (aOR = 2.9, 95% CI: 1.4–6.2, p < 0.01). Birds sampled from household farms had higher odds of AIV positivity than those sampled from LBMs (aOR = 2.8, 95% CI: 1.2–6.5, p = 0.02) (Table 3). Species-specific multivariable logistic regression analyses were conducted separately for quails and pigeons using the same explanatory variables, except species. In quails, clinically sick birds had 2.9 times higher odds of AIV positivity than healthy birds (p = 0.01). Birds from commercial farms had lower odds of AIV positivity compared with birds from LBMs (aOR = 0.1, 95% CI: 0.0–0.5, p = 0.01) (S1 Table). In pigeons, none of the investigated variables were significantly associated with AIV positivity, although the direction of the associations was generally consistent with the overall model (S2 Table).

**Table 3:** Multivariable logistic regression analysis of factors associated with AIV positivity in pigeons and quails.

Variable	Category	aOR	95% CI	p-value
Species	Pigeons	Reference	–	–
	Quails	4.1	1.8–10.6	<0.01*
Health condition	Healthy	Reference	–	–
	Sick	2.9	1.4–6.2	<0.01*
Source of sample	LBMs	Reference	–	–
	Commercial Farms	0.4	0.1–0.9	0.03*
	Household Farms	2.8	1.2–6.5	0.02*

* p-value < 0.05: statistically significant

## Discussion

This study demonstrates a notable prevalence of AIV in pigeons and quails in Chattogram, Bangladesh, underscoring their potential role in the transmission of AIV across diverse production and marketing systems. We identified an overall AIV prevalence of 14.3%, with subtype H9 predominating, consistent with the widespread circulation of low pathogenic AIVs in South Asian poultry systems. The significantly higher prevalence observed in quails (18.2%) compared with pigeons (7.4%) is consistent with previous studies in Bangladesh that report increased susceptibility of quails to AIV infection, potentially driven by intensive production practices and suboptimal biosecurity measures [40]. Our findings further support the hypothesis that quails may act as amplifying hosts for AIV, facilitating virus persistence and spread within mixed poultry systems, particularly under high-density farming and housing conditions [41,42]. Collectively, these results underscore the importance of integrating pigeons and quails, often excluded from routine surveillance, into national avian influenza monitoring and control frameworks to improve early detection and mitigate the risk of onward transmission.

The predominance of the H9 subtype detected in this study (11.3%) reflects the endemic circulation of this LPAI within the region, which increases the possibility of reassortment into more virulent strains [43]. This aligns with previous national surveillance reporting, which has shown H9N2 prevalence of 6–20% in quail and up to 5% in pigeons, highlighting the persistent risk posed by this subtype since 2015 [44–46]. The elevated prevalence of H9 in quail further suggests that this species may facilitate ongoing transmission cycles in household and market settings, as documented in Iran and Egypt, where H9 prevalence was strongly linked to substandard biosecurity [47,48]. Given that H9N2 has zoonotic potential and has caused human infections elsewhere, the high prevalence of AIV in quail warrants serious public health attention.

Although rare, the detection of H5 subtype (0.9%) in pigeons is noteworthy because it indicates that HPAI strains continue to circulate in avian populations of Bangladesh. This finding is consistent with earlier studies, which suggested that pigeons, although traditionally considered resistant, may occasionally become infected and shed the virus as a reservoir [46,49]. Importantly, the absence of H5 detection in quail in this study, while divergent from some earlier findings reporting 15.0% positivity in quail [45], may reflect the seasonal differences or localized circulation patterns. No H7 subtype was detected in this study, corroborating previous surveillance data that suggest low circulation of this subtype in pet birds in Bangladesh, although H7N9 has been sporadically reported in the country’s poultry populations [44]. Additionally, the presence of HA-untyped (2.7%) suggests the possibility of divergent or low-titre strains, emphasizing the need for molecular characterization and sequencing in future investigations, as these HA-untyped strains may indicate undetected variants within the local AI populations [43].

This study identified several factors associated with AIV positivity. Clinically sick birds were nearly three times more likely to test positive for AIV than apparently healthy birds, consistent with previous studies linking clinical illness with increased viral shedding. Birds sampled from household farms had higher odds of AIV positivity than birds sampled from LBMs, while birds from commercial farms had lower odds, highlighting the potential influence of biosecurity and management practices [50]. Although age was not retained in the final multivariable model, previous studies have reported higher AIV prevalence among young and intensively reared quails [45,46].

The detection of both HPAI and LPAI subtypes in pet birds underscores their potential role as intermediaries facilitating the circulation of viruses among wild birds, poultry, and humans. In Bangladesh and other developing countries, key biosecurity lapses, including mingling sick and healthy birds, inadequate cage hygiene, unregulated contact with wild birds, and poor handwashing among handlers, are exacerbated by limited awareness, poor infrastructure, and lack of enforcement of hygiene standards [12]. Addressing these gaps through a One Health approach by integrating veterinary, public health, and environmental measures may be helpful to mitigate risks by educating owners, ensuring separation of sick birds, restricting wild bird access to cages, and providing affordable sanitation facilities [50]. A previous study reported that only a minority of pet bird owners practiced basic hygiene, such as handwashing after handling birds, despite well-established recommendations for preventing zoonotic transmission [51]. Strengthening community-based education campaigns, enforcing simple hygiene protocols, and promoting active engagement of stakeholders at all levels are essential One Health strategies to reduce the risks associated with AI and improve public health outcomes in Bangladesh and similar settings.

Vaccination may play an important role in managing H9N2 in quail and pigeon populations, given the substantial health risks they pose to surrounding poultry [52]. Current data indicating the significant economic burden of H9N2 illustrates the necessity for vaccination, particularly considering the impact of co-infection with other pathogens [52]. Enhancing vaccination protocols could significantly diminish both the prevalence of the virus in these species and the consequential health risks to poultry farms. Moreover, the availability of vaccines for poultry substantiates the urgent need for their adaptation and effective application in quail and pigeons, thereby contributing to overall biosecurity in poultry farming [30].

This study provides additional evidence on the role of quail and pigeons in the epidemiology of AIV in Bangladesh. The consistently higher prevalence of AIV in quail birds and even sporadic detection in pigeons underscores the necessity of AI surveillance in household and market settings [46]. We recommend adopting a One Health strategy, which integrates veterinary, human, and environmental health, to curb the risk of AI emergence and zoonotic transmission. Strengthening biosecurity, conducting targeted outreach to bird owners, and improving diagnostic capacity for subtyping and clade identification will be critical next steps. We also recommend taking initiative for an ongoing surveillance along with an awareness campaign on improved biosecurity practices for all sectors of the poultry ecosystem, including pet birds, to prevent the emergence of novel strains and mitigate the risk to public and animal health [53,54].

Finally, this study provides important evidence on the epidemiology of AIV in pigeons and quails across household farms, commercial farms, and LBMs in Bangladesh. Nevertheless, several limitations should be considered when interpreting the findings. The cross-sectional design of this study limits our ability to establish the temporal relationship between AIV infection and the characteristics examined; therefore, while the observed associations provide useful epidemiological insights, they should be interpreted with appropriate caution regarding causality. This may be particularly relevant to the observed association between clinical sickness and AIV positivity, as clinical sickness could also occur as a consequence of infection. The study was conducted in selected upazilas of Chattogram, and the findings may not be generalizable to all regions of Bangladesh. In addition, a precision level of ±7% was used for the sample size calculation to balance statistical precision with field feasibility. This may have resulted in slightly wider confidence intervals than the conventional ±5% precision. Furthermore, the sample size was determined separately for pigeons and quails and was not specifically powered to compare AIV prevalence across different production systems. Species-specific analysis reduced the sample size for each model, particularly among pigeons, resulting in wide confidence intervals and limited statistical power. Therefore, the absence of statistically significant associations in the pigeon-specific model does not necessarily indicate the absence of biological effects.

## Conclusion

This study demonstrates the continued circulation of AIV, including HPAI H5 and LPAI H9, among pigeons and quails in Chattogram. Quails exhibited a disproportionately higher prevalence of AIV, particularly the H9 subtype, indicating their potential epidemiological importance in maintaining H9 circulation. These findings suggest that the potential role of quail in H9 circulation should be considered when refining Bangladesh’s existing avian influenza surveillance and vaccination strategies. The presence of HA untyped AIVs and the higher AIV positivity observed among birds sampled from household farms further indicate persistent gaps in biosecurity and ongoing opportunities for virus circulation and evolution. These findings underscore the need for sustained and expanded surveillance of these poultry species, particularly quail and pigeons, to enable early detection of emerging AIV strains with panzootic and zoonotic potential. Given that AIV vaccination is already practiced in Bangladesh, the potential inclusion of quail and pigeons in targeted vaccination strategies, alongside strengthened biosecurity and risk-based surveillance, warrants further evaluation as part of the integrated AIV control program.

## Supporting information

S1 Table Species-specific multivariable logistic regression analyses. (DOCX)

S2 Table PLOS questionnaire on inclusivity in global research. (DOCX)

S3 Data Supplementary dataset. The study dataset used for the statistical analyses presented in this study.
(XLSX)
