# Supplementary material for: Epidemiological characterization of avian influenza virus circulation in pigeons and quail across different production systems in Chattogram, Bangladesh
Source: PLoS One. 2026 Oct 26;21(10):e0361561. doi: 10.1371/journal.pone.0361561 (PMC13705595; doi:10.1371/journal.pone.0361561)
Supplement: Supplementary file 1 [file pone.0361561.s001.docx]

**Supplementary Table 1:** Multivariable adjusted association of AIV positivity with different factors in quail

| **Variable** | **Category** | **aOR** | **95% CI** | **p-value** |
| --- | --- | --- | --- | --- |
| Health condition | Healthy | Ref | - | - |
|  | Sick | 2.9 | 1.3 – 7.0 | **0.01*** |
| Source of sample | LBMs | Ref | - | - |
|  | Commercial Farms | 0.1 | 0.0 – 0.5 | **0.01*** |
|  | Household Farms | 2.2 | 0.8 – 6.1 | 0.12 |

**Supplementary Table 2:** Multivariable adjusted association of AIV positivity with different factors in Pigeon

| **Variable** | **Category** | **aOR** | **95% CI** | **p-value** |
| --- | --- | --- | --- | --- |
| Health condition | Healthy | Ref | - | - |
|  | Sick | 3.6 | 0.7 – 26.1 | 0.14 |
| Source of sample | LBMs | Ref | - | - |
|  | Commercial Farms | 0.5 | 0.0 – 5.2 | 0.54 |
|  | Household Farms | 2.3 | 0.4 – 17.1 | 0.36 |
