# Supplementary material for: Epidemiological characterization of avian influenza virus circulation in pigeons and quail across different production systems in Chattogram, Bangladesh
Source: PLoS One. 2026 Oct 26;21(10):e0361561. doi: 10.1371/journal.pone.0361561 (PMC13705595; doi:10.1371/journal.pone.0361561)
Supplement: Supplementary file 2 [file pone.0361561.s002.docx]

Inclusivity in global research

PLOS’ policy on inclusivity in global research aims to improve transparency in the reporting of research performed outside of researchers’ own country or community and ensures that PLOS publications reporting global research adhere to high standards for research ethics and authorship. Authors of relevant research articles may be asked to complete the questionnaire below, which outlines ethical, cultural, and scientific considerations specific to inclusivity in global research. This questionnaire may be requested when researchers have travelled to a different country to conduct research, if research uses samples collected in another country, research with Indigenous populations or their lands, or if research is on cultural artefacts. Researchers travelling to another country solely to use laboratory equipment will not normally be required to complete the questionnaire. However, the questionnaire can be requested at the journal’s discretion for any submission – if you have been requested to complete this questionnaire by the PLOS journal you submitted to, please do so.

Please complete the questionnaire below and include this as a Supporting Information file with your manuscript. Note that if your paper is accepted for publication, this checklist will be published with your article in the supporting information files. Please ensure that you reference the checklist in the main body of your manuscript. We suggest adding a subsection ‘Inclusivity in global research’ to your Methods section and adding the following sentence: “Additional information regarding the ethical, cultural, and scientific considerations specific to inclusivity in global research is included in the Supporting Information (SX Checklist)”

The questions have been designed to be applicable to a wide range of study types, and there are subsections for both human subjects research and non-human subjects research. If any of the questions are not relevant to your research please mark them as “N/A” as appropriate.

**Ethical considerations, permits and authorship**

*This section is applicable to all research types.*

Provide details as to who granted permissions and/or consent for the study to take place in the Methods section of your manuscript. This should include the names of **all** ethics boards, governmental organizations, community leaders or other bodies that provided approval for the study. If individuals provided approval refer to these people by their role or title but do not list their name(s).

Ethical approval for this study was granted by the Institutional Ethics Committee of Chattogram Veterinary and Animal Sciences University (CVASU) under protocol number CVASU/Dir/(R&E)EC/2019/126(1), dated December 29, 2019. The CVASU Ethics Committee comprises experts in human health, animal welfare, and environmental sciences, and provided comprehensive approval covering all human, animal, and environmental aspects of this research protocol. Written informed consent was obtained from all bird owners and market traders prior to sample collection and data collection activities. All procedures involving the collection of biological swab samples from pigeons and quail were conducted by trained field veterinarian under appropriate physical restraint to ensure the safety of both the birds and the handlers. The study objectives and procedures were clearly explained to the bird owners, market traders and written informed consent was obtained to grant permission for sampling of farmed birds and birds collected from live bird markets. All details are reported in the Methods section of the manuscript under the "Ethical Approval" subsection.

If there were any deviations from the study protocol after approval was obtained please provide details of these changes in the Methods section of your manuscript.
Did this study involve local collaborators that are residents of the country where the research was conducted or members of the community studied? If you do not have any authors from said communities, please provide an explanation for this below.

Yes, all authors of this study are affiliated with institutions in Bangladesh where the research was conducted.The study was conducted in collaboration with researchers from Chattogram Veterinary and Animal Sciences University (CVASU) and the Institute of Epidemiology, Disease Control and Research (IEDCR), Dhaka, Bangladesh. Individuals such as farmers and market traders contributed by providing information and access to birds; however, they did not meet PLOS authorship criteria, as their involvement was limited to participation and information provision. Their contributions are therefore acknowledged appropriately.

There were no deviations from the approved study protocol occurred during the research.

Everyone listed as an author should meet PLOS’ criteria for authorship and all individuals who meet these criteria should be included in the author byline, rather than the acknowledgements. For further information please see the journal’s Authorship Policy.

All individuals who contributed substantially to the study design, data collection, analysis, and manuscript preparation were included as authors in accordance with PLOS authorship guidelines.

**Human subjects research (e.g. health research, medical research, cross-cultural psychology)**

Did you obtain written informed consent from a representative of the local community or region before the research took place? How did you establish who speaks for the community? Details of written informed consent obtained from study participants should be reported separately in the Methods section of your manuscript.

This study was conducted in accordance with the human subject research guidelines set forth in the Declaration of Helsinki (1964) and its subsequent amendments . Written informed consent was obtained from individual bird owners and market traders for bird sampling and collection of farm-level and management data. These individuals were the appropriate representatives for their own farms and market stalls. Consent procedures are detailed in the Methods section of the manuscript. The study protocol was reviewed and approved by the Institutional Ethics Committee of Chattogram Veterinary and Animal Sciences University (CVASU). This study focused on avian influenza research in birds. However, we collected questionnaire data from bird owners and market traders regarding bird management practices and biosecurity measures.

How did members of the local community provide input on the aims of the research investigation, its methodology, and its anticipated outcome(s)?

The study was conducted by local Bangladeshi researchers who are members of the veterinary and public health community in Bangladesh. Research aims and methodology were developed in consultation with local veterinary authorities and were informed by national priorities for avian influenza surveillance and control. While bird owners and market traders did not participate in research design, they were fully informed about study objectives and provided voluntary consent to participate. Their input during data collection helped refine our understanding of local poultry management practices.

When engaging with the local community, how did you ensure that the informed consent documents and other materials could be understood by local stakeholders?

All consent procedures and data collection activities were conducted in Bengali, the local language, by trained field veterinarians who are native Bengali speakers. Study objectives, sampling procedures, and participant rights were clearly explained to bird owners and traders in simple, accessible language appropriate for individuals with varying educational backgrounds. Participants were given opportunities to ask questions and could withdraw at any time.

Will the findings of the research be made available in an understandable format to stakeholders in the community where the study was conducted (e.g. via a presentation, summary report, copies of publications, etc.)? Please provide details of how this will be achieved.

Yes. Study findings will be shared with relevant Bangladeshi authorities including the Department of Livestock Services, local veterinary offices, and poultry industry stakeholders. Results will be disseminated through open-access publication in PLOS ONE, making findings freely available to all stakeholders including farmers, veterinarians, market traders, and public health officials in Bangladesh. We will also prepare summary reports in Bengali for distribution to participating farms and markets through local veterinary channels.

**Non-human subjects research using specimens/ animals collected as part of the study, or those housed in archival collections. Examples include archaeology, paleontology, botany and zoology.**

Did the permission you obtained from a local authority to perform the study include an agreement on access to outputs and benefit sharing? This may include procedures to enable fair distribution of the benefits and resources arising from the research performed. Please include any details of Prior Informed Consent and Benefit Sharing Agreements obtained. These may be required by field-specific regulations, for example the Convention on Biological Diversity (CBD) and the associated Nagoya Protocol.

This study involved non-human animal sampling (pigeons and quails) conducted under institutional ethical approval. No formal agreements on access to research outputs or benefit sharing were established, as the study focused on epidemiological surveillance and management-related data collection. Formal benefit-sharing frameworks, including those under the Convention on Biological Diversity and the Nagoya Protocol, were not applicable to this research.

The ethical approval obtained from the Institutional Ethics Committee of Chattogram Veterinary and Animal Sciences University (CVASU) included authorization for sample collection and data generation within Bangladesh. As this research was conducted entirely by Bangladeshi researchers within Bangladesh using biological samples collected from domesticated birds (pigeons and quail) in Bangladesh, the research falls under national jurisdiction. All biological samples were collected, processed, analyzed, and stored within Bangladesh. No samples or materials were exported from the country. All research outputs and benefits remain with Bangladeshi institutions and researchers. Written consent for bird sampling was obtained from all bird owners and market traders, ensuring local stakeholders were informed participants in the research process.

If the material used in your study was imported, please A) provide the year it was imported and B) indicate whether permits were obtained to import/export the materials used, C) provide details of any permits obtained. If this information is not available, please indicate this.

No biological materials were imported or exported for this study. All biological specimens were collected in within Bangladesh from domesticated birds (quail and pigeons) local production systems and all samples remioaned within Bangladesh for processing and analysis

If you used archival specimens, please state how the material used in your study was acquired by the institute it is held in and provide details of any permits obtained for the original excavations/ sample collection. If this information is not available, please indicate this.

This study did not use archival specimens. All biological samples (cloacal and oropharyngeal swabs) were collected prospectively between November 26, 2021, and April 2, 2022, from live birds with written consent from their owners.

How was the potential cultural significance of the materials collected in your study to local communities considered in your research design? Were Indigenous peoples and/or local researchers and institutions involved with archaeological excavations / collection of specimens? If so, please provide a description of their involvement.

This study involved collection of biological swabs from domesticated birds (pigeons and quail) for disease surveillance purposes and does not involve materials with cultural or archaeological significance. The study did not involve the collection of cultural artefacts or materials of cultural significance. Therefore, cultural heritage considerations were not applicable to the study design.

If your manuscript includes photographs of human remains please indicate whether authors obtained permission from descendants or affiliated cultural communities to do so.

The manuscript does not include photographs of human remains.
